# Molecular mechanism of ensitrelvir inhibiting SARS-CoV-2 main protease and its variants

**DOI:** 10.1038/s42003-023-05071-y

**Published:** 2023-07-05

**Authors:** Mengmeng Lin, Xudong Zeng, Yinkai Duan, Zinan Yang, Yuanyuan Ma, Haitao Yang, Xiuna Yang, Xiang Liu

**Affiliations:** 1 College of Life Sciences, State Key Laboratory of Medicinal Chemical Biology, Nankai University, Tianjin, China; 2 Institute of Life Sciences, Chongqing Medical University, Chongqing, China; 3 Shanghai Institute for Advanced Immunochemical Studies and School of Life Science and Technology, ShanghaiTech University, Shanghai, China

**Keywords:** X-ray crystallography, Structural biology

## Abstract

SARS-CoV-2 poses an unprecedented threat to the world as the causative agent of the COVID-19 pandemic. Among a handful of therapeutics developed for the prevention and treatment of SARS-CoV-2 infection, ensitrelvir is the first noncovalent and nonpeptide oral inhibitor targeting the main protease (M^pro^) of SARS-CoV-2, which recently received emergency regulatory approval in Japan. Here we determined a 1.8-Å structure of M^pro^ in complex with ensitrelvir, which revealed that ensitrelvir targets the substrate-binding pocket of M^pro^, specifically recognizing its S1, S2, and S1' subsites. Further, our comprehensive biochemical and structural data have demonstrated that even though ensitrelvir and nirmatrelvir (an FDA-approved drug) belong to different types of M^pro^ inhibitors, both of them remain to be effective against M^pro^s from all five SARS-CoV-2 variants of concern, suggesting M^pro^ is a bona fide broad-spectrum target. The molecular mechanisms uncovered in this study provide basis for future inhibitor design.

## Abstract (web summary)

Structural and biochemical characterization of the SARS-CoV-2 main protease in complex with inhibitor ensitrelvir or nirmatrelvir reveals the mechanisms of inhibition.

## Introduction

SARS-CoV-2 poses an unprecedented threat to the world as the causative agent of the COVID-19 pandemic. Currently, a handful of therapeutics have been approved for the prevention and treatment of SARS-CoV-2 infection, including vaccinations, monoclonal antibodies, and compounds that target key enzymes with crucial roles in the viral life cycle^1,2^. However, the long-lasting pandemic plus the error-prone nature of the RNA viral genome enables SARS-CoV-2 to accumulate a variety of mutations, giving rise to various mutant strains that could potentially impair the efficacy of existing therapies^3,4^. The World Health Organization (WHO) named COVID-19 variants with increased transmissibility or harmful changes in epidemiology variants of concern (VOCs). By March 2023, the WHO had identified five VOCs, including Alpha (B.1.1.7), Beta (B.1.351), Gamma (P.1), Delta (B.1.617.2), and Omicron (B.1.1.529). Since May 2021, these variants have become the dominant variants in more than 90 countries^5,6^.

M^pro^, also named 3-chymotrypsin-like protease (3CLpro), is a viral-encoded cysteine protease that plays a fundamental role in viral replication. After invading the host cell, the positive-sense single-stranded viral RNA genome will be treated as mRNA by the host ribosome and generate two long polyproteins named pp1a and pp1ab, which will be further proteolytically processed and give rise to the various non-structural proteins (nsps) required for the subsequent viral life cycle^7^. During the proteolytic process of pp1a and pp1ab, M^pro^ is indispensable and cleaves polyproteins at no less than 11 conserved sites. Owing to the key role of the protein in viral replication, two oral antiviral drugs targeting M^pro^ received emergency use authorization (EUA): nirmatrelvir and ensitrelvir. Nirmatrelvir is the main ingredient of Paxlovid, developed by Pfizer, which has been officially approved by the FDA^8^. Paxlovid showed a greater reduction in the risk of hospitalization and death than a placebo. Ensitrelvir is the first oral noncovalent, nonpeptide inhibitor developed by Shionogi^9^. Unlike nirmatrelvir, ensitrelvir does not need pharmaceutical boosters such as ritonavir and can be directly used to treat patients with mild COVID-19. Ensitrelvir received emergency authorization in Japan after treatment showed rapid SARS-CoV-2 clearance in a phase 2/3 clinical trial and was well tolerated^10^. Last month, ensitrelvir just received Phase III clinical approval in the United States. It is critical to understand its appropriate molecular mechanism of inhibition. Besides, SARS-CoV-2 VOCs carry mutations at varying frequencies in the M^pro^ that are specific to Alpha, Beta, Gamma, Lambda, and Omicron. where Alpha, Beta, and Gamma have the substitution K90R, Lambda has the substitution G15S, and Omicron has the substitution P132H^11^. It remains unclear whether these changes in M^pro^ across different SARS-CoV-2 variants will affect the architecture of the reaction pocket and thus affect the inhibition of current compounds targeting M^pro^.

In this study, we combined the enzymatic activity assay and crystallography to study the structure-activity relationship (SAR) between SARS-CoV-2 M^pro^ and the inhibitors, respectively. We obtained a total of 10 crystal structures of M^pro^, including the apo-forms of the variants and M^pro^/variant-inhibitor complexes. Our results presented here will facilitate future antiviral design.

## Results

### Ensitrelvir is a potent inhibitor against WT SARS-CoV-2 M^pro^

**Fig. 1:**
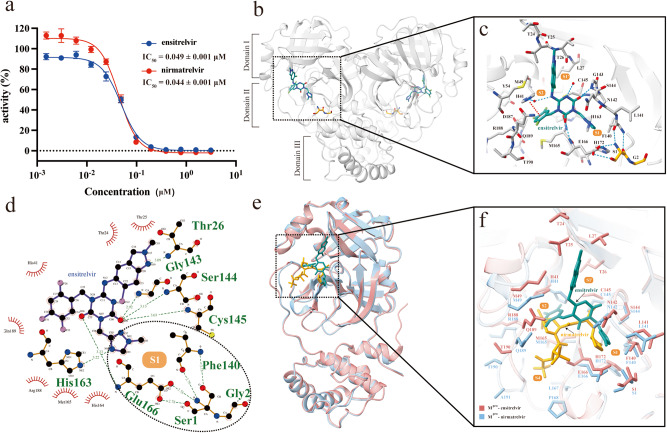
Comparison of in vitro enzyme activity inhibition and molecular mechanisms between ensitrelvir and nirmatrelvir. **a** The IC_50_ of ensitrelvir and nirmatrelvir against WT SARS-CoV-2 M^pro^. Data points are shown as the mean ± SD (*n* = 3). **b** The crystal structure of dimeric M^pro^ complex with ensitrelvir. **c** The interaction network of ensitrelvir in the substrate-binding pocket of M^pro^. Ensitrelvir is shown in green, M^pro^ is shown in silver, and the residues of the neighboring protomer are labeled in yellow. Blue dotted lines represent hydrogen bonds, red dashed lines represent π-π stack, and red spheres represent water molecules. The substrate pocket is indicated separately. **d** Topology of the hydrogen-bond network between ensitrelvir and M^pro^. Hydrogen bonds are indicated by green dashed lines, and dashed boxes indicate interaction with the stable S1 pocket. **e** Band diagram of the WT and ensitrelvir complex crystal structure superimposed on WT bond nirmatrelvir. **f** Analysis of the residues involved in the interactions between ensitrelvir and nirmatrelvir in the substrate-binding pocket. Ensitrelvir is highlighted in green, while nirmatrelvir is highlighted in yellow.

Based on the fluorescence-resonance-energy transfer (FRET)-based assay we previously established, the inhibition of ensitrelvir on SARS-CoV-2 M^pro^ was performed to examine the enzyme kinetics of WT. The half-maximal inhibitory concentrations (IC_50_) value of ensitrelvir against SARS-CoV-2 M^pro^ is 0.049 μM (Fig. 1a), while the IC_50_ of nirmatrelvir against WT is 0.044 μM, indicating that ensitrelvir exhibited as potent inhibition on viral protease as nirmatrelvir did in vitro.

### The crystal structure of SARS-CoV-2 M^pro^ in complex with ensitrelvir

In order to elucidate the molecular inhibition mechanism of ensitrelvir, we determined a 1.8-Å M^pro^-ensitrelvir complex structure (PDB: 8HBK). In the crystals, there is merely one protomer in an asymmetric unit, and all 1–301 residues could be traced in the electron density map. Two protomers form a functional dimer by a crystallographic twofold axis of symmetry. The overall protomer of M^pro^ comprises three domains (Fig. 1b). Domain I (residues 8–101) and domain II (residues 102–184) possess an antiparallel β-barrel structure and take on a chymotrypsin-like fold, harboring the catalytic pocket between them. Domain III (residues 201–303) is a globular domain composed of five antiparallel α-helixes unique to M^pro^ and is crucial for the dimerization of M^pro^, which is a prerequisite for its catalytic activity. The catalytic pocket containing the Cys-His dyad is located in the cleft between domain I and domain II (Fig. 1b). An ensitrelvir molecule could be identified in the substrate-binding pocket of each protomer, occupying S1, S2, and S1' subsites of M^pro^. In the crystal structure of dimeric M^pro^, the N-terminus of one promoter deeply inserts into the S1 subsite of the neighboring promoter and participates in the stabilization of the substrate pocket (Fig. 1c). In the S1 subsite, the Ser1 from the neighboring protomer stabilizes the pocket by forming four hydrogen bonds with E166 and F140. The 1-methyl-1*H*-1,2,4-triazole group forms a hydrogen bond with the sidechain imidazole group of H163. In the S2 subsite, the 2,4,5-trifluoromethyl forms a π-π stack with the sidechain of H41. In the S1' subsite, the 6-chloro-2-methyl-2*H*-indazole part interacts with the NH of the mainchain of T26 through a hydrogen bond. In addition, H163, C145, G143, and Q189 are also involved in the hydrogen-bond network to stabilize the binding of ensitrelvir (Fig. 1d). Nirmatrelvir forms a covalent bond with C145 of M^pro^ in the S1' subsite, whereas ensitrelvir forms no covalent bond in the substrate pocket. Instead, it interacts through hydrogen bonding with C145 and T26 in the S1' pocket. Furthermore, nirmatrelvir stabilizes in the S4 pocket through extensive hydrophobic interactions, whereas ensitrelvir has few interactions in this pocket. The interaction network in the S1 subsite is highly conserved for both inhibitors, as they both stabilize through hydrogen bonds with E166, F140, Ser1, and H172 in the S1 pocket (Fig. 1e, f).

### The G15S, K90R, and P132H substitutions have a limited impact on the structural and enzymatic properties of M^pro^

To elaborate on the impact of the G15S, K90R, and P132H mutations on the structure of M^pro^, the apo-form structures of G15S, K90R, and P132H were determined at 1.77, 1.66, and 1.82 Å resolution, respectively (Supplementary Table S1). There is only one protomer in an asymmetric unit in each crystal structure, and all 1–301 residues could be traced in the electron density map.

**Fig. 2:**
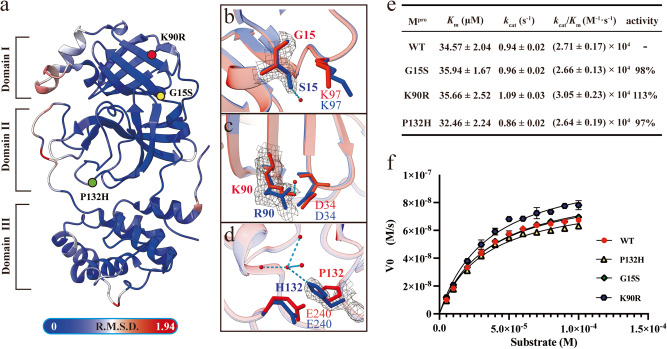
Comparison of the effects of amino acid substitutions in the SARS-CoV-2 variants on the structural and in vitro enzymatic activity properties. **a** Superposition of crystal structures of WT, G15S, K90R, and P132H. The color spectrum indicates the RMSD of the Cα atoms from the aligned structures. The position of residue 15 is shown as a yellow circle, 90 is shown as a red circle, and 132 is shown as a green circle. **b**–**d** Comparison of the region in the vicinity of residues 15, 90, and 132 between WT and G15S, K90R, and P132H, respectively. WT is shown in marine blue, and G15S, K90R, and P132H are shown in salmon. The *2Fo-Fc* density map is shown as a black grid contour at 1.0σ. **e** Enumerated enzyme activity data of G15S, K90R, P132H, and WT calculated by the Michaelis equation, including *K*m, *k*cat, and *k*cat/*K*m. **f** Characterization of enzymatic activity shows that G15S, K90R, and P132H display very similar activity to WT. Data points are shown as the mean ± SD (*n* = 3).

The mutant residues 15 and 90 are located in domain I and residue 132 is located in domain II. All these mutations are far away from the catalytic pocket (Fig. 2a and Supplementary Fig. S3); thus, it is reasonable to speculate that the G15S, K90R, and P132H mutations do not directly affect the architecture of the catalytic pocket. This was further confirmed when comparing the G15S, K90R, and P132H structures with the M^pro^ apo-form structure (PDB ID: 6Y2E). As shown in Fig. 2a, G15S, K90R, and P132H showed merely a slight difference from WT, with average root-mean-square deviation (RMSD) values of only 0.54 Å, 0.52 Å, and 0.58 Å, respectively, indicating that all these three mutations had a limited impact on the apo structure of M^pro^. It is worth mentioning that the mutation of the amino acid at position 15 from glycine to serine did not cause any serious structural changes to the backbone, except that the substitute of serine formed an additional hydrogen bond with a surrounding water molecule (Fig. 2b). The mutation of the amino acid at position 90 from lysine to arginine results in the breaking of a hydrogen bond from lysine to the water molecule but does not significantly alter the conformations of surrounding residues (Fig. 2c). Similarly, the P132H substitution did not change the architecture of the mainchain at this site or the residues in the vicinity except that the sidechain of E240 was slightly “pushed” away from residue 132. It is presumably caused by the steric hindrance of the histidine, and it is noticed that the sidechain of H132 formed an extra hydrogen bond with a water molecule (Fig. 2d).

To further validate whether these slight structural variations affect enzymatic efficiency, a previously established fluorescence-resonance-energy transfer (FRET)-based assay was used to compare the enzyme kinetic parameters of WT, G15S, K90R, and P132H. As shown in Fig. 2e, f, the catalytic efficiency (*k*_cat_/*K*_m_) values were determined to be 2.66 × 10^4 ^M^−1^s^−1^ for G15S, 3.05 × 10^4 ^M^−1^s^−1^ for K90R, 2.64 × 10^4 ^M^−1^s^−1^ for P132H, respectively, which are comparable to 2.71 × 10^4 ^M^−1^s^−1^ for WT. It is indicated that the G15S, K90R, P132H, and WT have similar enzymatic kinetic parameters and the impact of the three substitutions on M^pro^ could be neglected.

### Ensitrelvir remains strong inhibition on SARS-CoV-2 M^pro^ variants

**Fig. 3:**
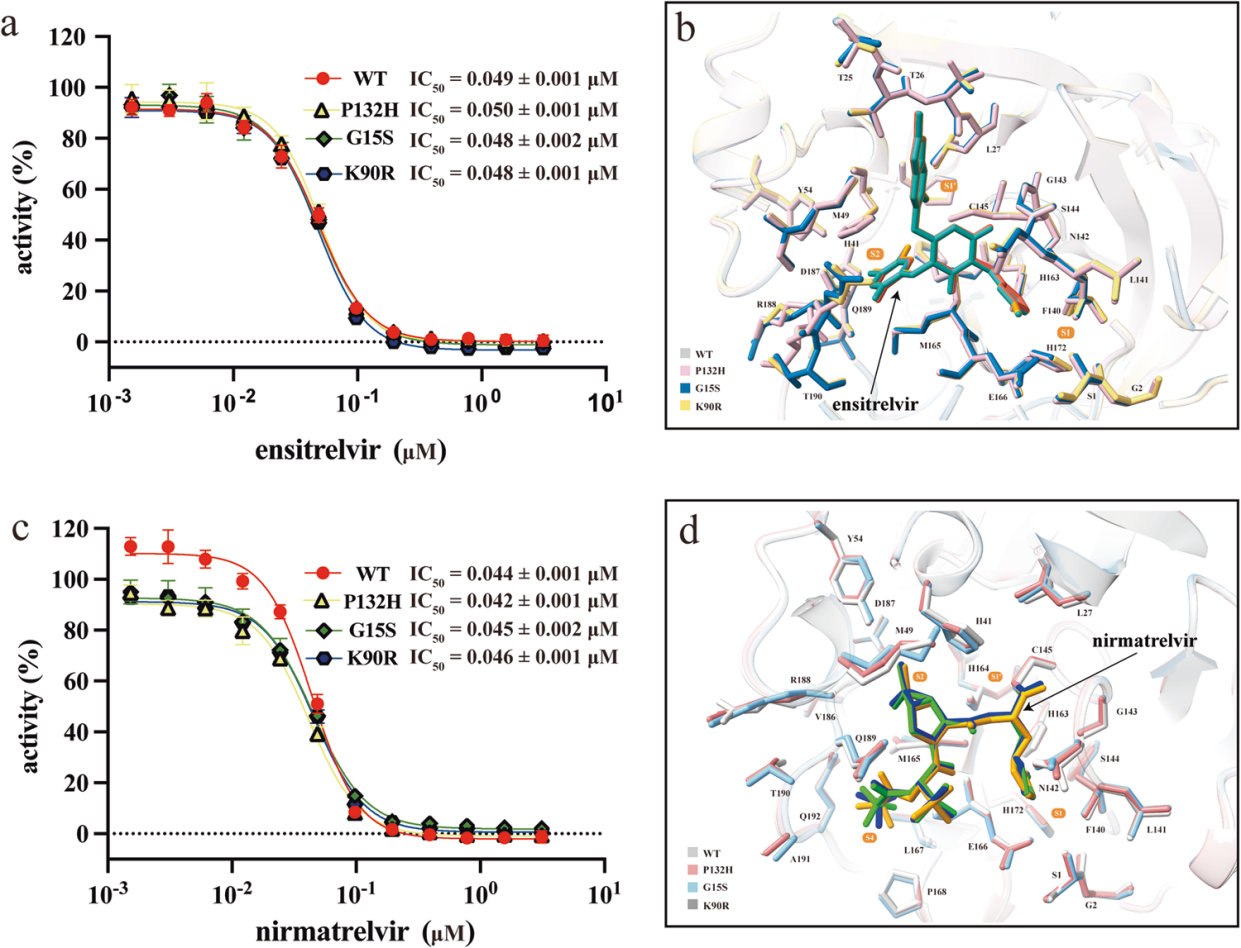
Structural basis for in vitro inhibition of SARS-CoV-2 variants by ensitrelvir and nirmatrelvir. **a**, **c** The IC_50_ of ensitrelvir and nirmatrelvir against M^pro^ and variants, respectively. Data points are shown as the mean ± SD (*n* = 3). **b**, **d** Superimposed crystal structures of WT M^pro^ and variants complexed with ensitrelvir and nirmatrelvir, respectively. The key residues in the pocket are shown in sticks.

Next, we tested the half-maximal inhibitory concentrations (IC_50_) of ensitrelvir against SARS-CoV-2 M^pro^ and variants. The results showed that ensitrelvir exhibited similar inhibition on WT and its variants with an IC_50_ of approximately 0.04 μM (Fig. 3a). To obtain the structural basis for the inhibition of SARS-CoV-2 M^pro^ and variants of the ensitrelvir, we eventually determined the structures of three variants individually with ensitrelvir for a total of three complex structures (Supplementary Table S1). Superimposition of the structures of all three complexes has shown that all three variant mutant sites (G15, K90, P132) are more than 20 Å away from the binding sites of ensitrelvir (Fig. 2a and Supplementary Fig. S3), indicating that these changes in M^pro^ between different SARS-CoV-2 variants may not affect the architecture of the substrate-binding pocket and thus would not impair the efficacy of the current compounds targeting M^pro^. Among the structures of mutant complexes of ensitrelvir, H163, E166, C145, G143, and T26 are involved in hydrogen bonding (Fig. 3b), and certain residues in the substrate-binding pocket participate in stabilizing ensitrelvir, which has demonstrated a conservative binding mode among M^pro^ from the WT and other variants. To summarize, all the data obtained above strongly support that M^pro^ from SARS-CoV-2 WT and its variants have similar structural features and kinetic characters, and the G15S, K90R, and P132H substitutions do not impair the inhibition of ensitrelvir in vitro.

### Nirmatrelvir is also a potent inhibitor against SARS-CoV-2 M^pro^ variants

Nirmatrelvir is also an inhibitor targeting SARS-CoV-2 M^pro^, which has just been fully approved to treat mild to moderate COVID-19 in adults at risk of severe infections. Next, we tested IC_50_ values of nirmatrelvir against SARS-CoV-2 M^pro^ and variants to see whether these mutations affect nirmatrelvir binding. The results showed that IC_50_ of nirmatrelvir against WT was 0.044 μM while IC_50_ of ensitrelvir against WT was 0.049 μM (Fig. 3c), suggesting that nirmatrelvir strongly inhibits WT M^pro^ like ensitrelvir. In addition, the IC_50_ values of nirmatrelvir against all variants are approximately 0.04 μM, indicating that these mutations did not obviously affect nirmatrelvir binding.

To obtain the structural basis for the inhibition of SARS-CoV-2 M^pro^ and variants of nirmatrelvir, we eventually determined the structures of three variants individually with nirmatrelvir for a total of three complex structures (Supplementary Table S1). Superimposition of the structures of all three complexes has shown all three variant mutant sites (G15, K90, P132) were more than 20 Å away from the binding site of nirmatrelvir (Fig. 2a and Supplementary Fig. S3), indicating that these changes in M^pro^ would not impair the in vitro efficacy of nirmatrelvir. All three mutant M^pro^ structures show little difference from that of WT, with an average RMSD of only 0.25–0.35 Å, and neither the binding pose of the compound nor the conformation of residues participating in drug binding exhibits significant differences. As shown in our previous analysis of the complex structure of WT with nirmatrelvir (PDB ID: 7VH8)^12^, the nitrile group of nirmatrelvir is attached to the Sγ atom of C145 through a standard 1.8 Å C-S covalent bond, the classical (S)-γ-lactam ring at the P1 position fits into the S1' subsite, and a hydrogen bond is formed between the oxygen atom of the lactam ring and the Nε2 atom of H163. In addition, the Oε1 atom of E166 interacts with the NH group to stabilize nirmatrelvir. The rigid dimethylcyclopropyl proline (DMCP) located at the S2 subsite is surrounded by extensive hydrophobic interactions. Most of the amino acids used to stabilize nirmatrelvir near the substrate pocket described above show great similarity in the complex structures of M^pro^ (Fig. 3d), which may imply that nirmatrelvir may exhibit similar inhibition for WT M^pro^ and other variants, which is consistent with the in vitro enzyme activity inhibitory of nirmatrelvir (Fig. 3a, c).

## Discussion

In the 21st century, three previously unknown coronaviruses have spread globally, including severe acute respiratory syndrome (SARS) caused by SARS-CoV in 2003, Middle East respiratory syndrome (MERS) caused by MERS-CoV in 2012, and the current COVID-19 caused by SARS-CoV-2. During the first two rounds of the coronavirus outbreak, no approved targeted therapies, vaccines, or compounds were available for treatment^13^. However, for the COVID-19 pandemic, it is the first time newly developed numerous approved vaccines, targeted compounds, etc., that we can use to battle against the coronavirus^14,15^. Unfortunately, due to the long-term prevalence of the virus in the population worldwide and the error-prone nature of RNA viruses, an increasing number of SARS-CoV-2 variants have been reported worldwide, raising concerns about the effect of current vaccines and compounds.

M^pro^ has received much attention in the last three years as an ideal target for drugs against SARS-CoV-2. As the first noncovalent and non-peptidomimetic drug candidate^16^, ensitrelvir has just received Phase 3 clinical approval in the United States. It is critical to understand its molecular mechanism of inhibition. In this work, a 1.8-Å M^pro^-ensitrelvir complex structure (PDB: 8HBK) was determined to elucidate the precise molecular inhibition mechanism of ensitrelvir. We found that ensitrelvir mainly recognizes the S1, S2, and S1' subsites of M^pro^ and relies on the stability of the hydrogen bonding network in the substrate-binding pocket, unlike the covalent inhibition mechanism of nirmatrelvir. In addition, the functional M^pro^ exists in a homo-dimer form in the physiological state, and the N-terminus residues 1–7 penetrate deeply into the substrate-binding pocket of the neighboring promoter and the N-terminus serine residue stabilizes the S1 subsite by hydrogen bonding, contributing to the stability of the substrate pocket and ensitrelvir binding (Fig. 2c, d). This differs from the previously reported model (PDB ID:7VU6)^9^, in which the N-terminal of M^pro^ lacks two residues (Ser1 and Gly2), while these two N-terminal residues can be traced in our complex structure (PDB ID:8HBK) based on the clear electron density (Supplementary Fig. S1). Moreover, the electron density of the Serl and Gly2 at the N-terminus is also clearly visible in all the complex structures of nirmatrelvir we solved (Supplementary Fig. S2).

The disruption of the N-terminus of M^pro^ may result from their construction of in vitro preparations of the protein. In previous research, we identified that the N-terminus of M^pro^ of SARS had excess amino acids, which will significantly impact on in vitro enzyme activity. The enzyme activity of M^pro^ with two extra residues at the N-terminus (GS-M^pro^) decreased by about 24 times compared to the clean N-terminus M^pro^ ^17^. The additional amino acids (Gly-2 and Ser-1) may have led to a difference in M^pro^ activity compared to the physiological state, with the protein being less active in vitro. This may explain why ensitrelvir exhibits stronger inhibition of enzyme activity than nirmatrelvir in vitro in that work, while our results show that the enzyme activity inhibition of ensitrelvir is similar to that of nirmatrelvir. Furthermore, there is a big gap in binding affinity between the two types of proteins, and the *K*d of GS-M^pro^ was 418 and 8.43 nM compared to the clean N-terminus M^pro^ (Supplementary Fig. S4). Under physiological conditions, residues in the N-terminus of M^pro^ penetrate deep into the substrate pocket of the neighboring protomer and are involved in stabilizing the S1 subsite^18^. The extra residues may spatially block ensitrelvir from entering the substrate pocket.

Overall, the apo-form structures and in vitro enzyme activity assays of several variants suggest that minor changes in M^pro^ have little effect on the overall structure, particularly the substrate pocket and the active center, which is consistent with our earlier findings. In addition, mutations in variants do not alter the properties of M^pro^, so they may not impair the in vitro enzymatic inhibition of nirmatrelvir and ensitrelvir, which further suggests that M^pro^ is an ideal drug target because it is one of the least variable viral components. Finally, we determined the in vitro inhibitory effects of ensitrelvir and nirmatrelvir on M^pro^ enzymatic activities, and the results showed that ensitrelvir and nirmatrelvir exhibit consistent in vitro enzymatic inhibition against SARS-CoV-2 M^pro^ and its variants, which is consistent with their excellent clinical efficacy. Although these two inhibitors belong to different types, both of them remain to be effective against M^pro^s from all 5 SARS-CoV-2 variants of concern, suggesting M^pro^ is a bona fide broad-spectrum target.

Recently, a 2.2 Å resolution complex structure of M^pro^-ensitrelvir was reported^19^. In their functional dimer, one protomer has clear electron density for its N-terminus, but its neighboring protomer does not. In the 1.8 Å resolution structure in this work, clear electron density can be observed for M^pro^ N-terminus, and we provide an accurate model for elucidating the ensitrelvir binding mode. The molecular mechanisms uncovered in this study provide the basis for future inhibitor design.

## Methods

### Cloning, protein expression, and purification of SARS-CoV-2 variant M^pro^

The expression plasmid used to produce full-length M^pro^ was obtained by using site-directed mutagenesis to introduce G15S, K90R, and P132H substitutions into the expression plasmid of full-length WT M^pro^. The expression plasmid was transformed into *E. coli* BL21 (DE3) cells and then cultured in a 2-L shaking flask with 1 L Luria broth medium containing 0.1 g/L ampicillin at 37 °C. When the optical density at 600 nm of the bacteria reaches 0.6–0.8, a final concentration of IPTG was added to the culture to induce protein expression at 16 °C. After 10 h, the bacteria were pelleted by centrifugation at 3000×*g* for 15 min. Then the bacteria were resuspended in lysis buffer (50 mM HEPES, pH 8.0, 300 mM NaCl), and the supernatant was incubated with Ni-NTA agarose gravity column (GE) after centrifuging at 18,000 rpm for 30 min. The fusion proteins were washed with wash buffer (50 mM HEPES, pH 8.0, 300 mM NaCl, 20 mM imidazole). After washing 10–20 column volume, the PreScission protease was added to remove the His-tag. Then the samples of the protein were loaded onto a Hitrap Q HP column and then purified by size-exclusion chromatography with a Superdex 75 increase column with storage buffer (50 mM HEPES, pH 7.5, 150 mM NaCl, and 4 mM DTT). The fractions were concentrated to 10 mg/mL for the next test^20^.

### Crystallization, data collection, and structure determination

The crystals of M^pro^ apo forms were directly screened by crystallization kits, and the crystals of the complexes with inhibitors were screened through co-crystallization of the variants M^pro^ at a concentration of 5 mg/ml with nirmatrelvir or ensitrelvir at 0.5 mM. The crystals were obtained at 18 °C by the sitting method. Crystals were cryoprotected using the reservoir solution with 20% ethylene glycol and flash-frozen in liquid nitrogen. Diffraction data were collected at 100 K at a wavelength of 0.9785 Å. The structures were solved by molecular replacement with the program *CCP4* using the complex structure of WT M^pro^ and N3 (PDB ID: 7VH8) as a search model^12^. The model was refined using *PHENIX*^21^ and manually constructed using *Coot*^22^.

### IC_50_ measurement

The method of IC_50_ measurement has been previously demonstrated^20^. In brief, the fluorescent substrate was applied to measure the hydrolytic activity of M^pro^. M^pro^ (0.1 μM) was incubated with different concentrations of nirmatrelvir or ensitrelvir for 90 s before 10 μM substrate was added. Fluorescence intensity was monitored by an EnVision multimode plate reader (Perkin Elmer) using wavelengths of 320 nm for excitation and 405 nm for emission. The changes in initial rates when adding different concentrations of nirmatrelvir or ensitrelvir were calculated to evaluate the inhibitory effect. The dose-response curve for IC_50_ values was determined by nonlinear regression using GraphPad Prism.

### ITC assay

The ITC experiment was performed on a MicroCal PEAQ-ITC. After cleaning the sample cell and needle, the OM^pro^ (20 μM) protein was carefully injected into the sample cell using a micro-syringe without any bubbles, and ensitrelvir (200 μM) was filled into a 40 μL titration syringe. OM^pro^ and ensitrelvir were diluted with TB (1% DMSO, pH = 8.0), and deionized water was injected into the reference cell as a heat balance control. After 35 times titrations of ensitrelvir into the sample cell at a constant rate of 150 s, the One Set of Sites is selected as the fitting model.

### Statistics and reproducibility

Statistical analysis was carried out using Prism software. The amount of enzyme was determined by the initial experiment, and the enzyme kinetic and half-inhibition tests consisted of three replicate trials.

### Reporting summary

Further information on research design is available in the Nature Portfolio Reporting Summary linked to this article.

## Supplementary information

Supplementary Information

Description of Additional Supplementary Files

Supplementary Data 1

Reporting Summary

## Data Availability

All experimental data are provided in the manuscript. The structures determined in this study have been deposited to the Protein Data Bank (PDB) under accession codes: 8HBK (WT-Ensitrelvir), 8HOM (P132H-Ensitrelvir), 8INX (G15S-Ensitrelvir), 8INY (K90R-Ensitrelvir), 8HOZ (P132H-Nirmatrelvir), 8INU (G15S-Nirmatrelvir), 8INW (K90R-Nirmatrelvir), 8HOL (P132H), 8INQ (G15S), 8INT (K90R). The structures of the M^pro^-nirmatrelvir complex and M^pro^-Ensitrelvir complex were downloaded from PDB under accession codes 7VH8 and 7VU6, respectively. Source data for figures can be found in Supplementary Data 1.

## Materials availability

Materials used in this study will be made available under an appropriate Materials Transfer Agreement.

## Code availability

No code was used for this study.
