# Supplementary material for: Molecular mechanism of ensitrelvir inhibiting SARS-CoV-2 main protease and its variants
Source: Commun Biol. 2023 Jul 5;6:694. doi: 10.1038/s42003-023-05071-y (PMC10322880; doi:10.1038/s42003-023-05071-y)
Supplement: Supplementary file 1 — Supplementary Information [file 42003_2023_5071_MOESM1_ESM.pdf]

Supplementary information for

Molecular mechanism of ensitrelvir inhibiting SARS-CoV-2 main protease and its variants

Mengmeng Lin[1,2,3], Xudong Zeng[3], Yinkai Duan[3], Zinan Yang[3], Yuanyuan Ma[3], Haitao Yang[3], Xiuna Yang[3,*], Xiang Liu[1,*]

[1]College of Life Sciences, State Key Laboratory of Medicinal Chemical Biology, Nankai University, Tianjin, China

[2]Institute of Life Sciences, Chongqing Medical University, Chongqing, China

[3]Shanghai Institute for Advanced Immunochemical Studies and School of Life Science and Technology, ShanghaiTech University, Shanghai, China.

***corresponding authors**

Email: liux@nankai.edu.cn; yangxn@shanghaitech.edu.cn

Contents

Table S1 Data collection and refinement statistics S2

Figure S1 Superimposed structures of WT M^{pro} and variants complexed with ensitrelvir in the dimer S5

Figure S2 Superimposed structures of WT M^{pro} and variants complexed with nirmatrelvir in the dimer S5

Figure S3 2*Fo-Fc* map of M^{pro}-compound complex structures. S6

Figure S4 Affinity testing of ensitrelvir with M^{pro} and GS-M^{pro}. S7

Table S1. Data collection and refinement statistics.

	M^{pro} (P132H)-apo	M^{pro} (G15S)-apo	M^{pro} (K90R)-apo
	PDB ID: 8HOL	PDB ID: 8INQ	PDB ID: 8INT
**Data Collection**			
Space group	*C*2	*C*2	*C*2
Wavelength (Å)	0.9792	0.9537	0.9537
Cell dimensions			
a, b, c (Å)	97.702, 81.615, 51.72	97.507, 80.562, 51.548	97.118, 81.021, 51.486
α, β, γ (°)	90, 114.807, 90	90, 114.51, 90	90 ,114.92, 90
Resolution (Å)	42.29-1.82 (1.93-1.82)[a]	29.44-1.77 (1.83-1.77)	29.41-1.66 (1.72-1.66)
No. of unique reflections	32849 (4976)	35266 (5628)	42299 (6755)
Completeness (%)	98.5 (92.5)	99.5 (98.6)	99.6 (99.3)
R_{merge} (%)[b]	8.5 (90.8)	8.0 (95.1)	4.8 (87.3)
Mean $I/\sigma I$	13.43 (1.73)	13.60 (2.03)	18.77 (1.84)
$CC_{1/2}$ [c]	99.8 (81.2)	99.9 (84.7)	100.0 (85.9)
Redundancy	3.8 (3.7)	6.9 (5.6)	6.9 (6.3)
Wilson B factors (Å^2)	32.48	27.63	28.85
**Refinement**			
Resolution (Å)	42.29-1.82	29.44-1.771	29.41-1.66
No. of reflections used	32817	35239	42274
R_{work} / R_{free}[d]	18.31/21.53	19.68/22.31	20.24/22.66
No. atoms			
Protein	2485	2312	2319
Ligand/ion	0	0	0
Water	141	162	177
B-factors (Å^2)			
Protein	43.26	34.30	38.89
Ligand/ion	0	0	0
Water	45.90	38.82	45.65
R.m.s. deviations			
Bond lengths (Å)	0.008	0.007	0.005
Bond angles (°)	0.98	0.98	0.89
Ramachandran plot (%)			
Favoured (%)	97.69	97.99	97.99
Allowed (%)	2.31	2.01	2.01
Outliers (%)	0	0	0

	M^{pro} (P132H) - nirmatrelvir	M^{pro} (G15S) - nirmatrelvir	M^{pro} (K90R) - nirmatrelvir
	PDB ID: 8HOZ	PDB ID: 8INU	PDB ID: 8INW
**Data Collection**			
Space group	$P\,2_1\,2_1\,2$	$P\,2_1\,2_1\,2$	$P\,2_1\,2_1\,2$
Wavelength (Å)	0.9792	0.9537	0.9537
Cell dimensions			
a, b, c (Å)	45.667, 63.546, 104.671	45.604, 63.769, 105.672	45.627, 64.033, 105.14
α, β, γ (°)	90, 90, 90	90, 90, 90	90, 90, 90
Resolution (Å)	54.32-2.83 (3.0-2.83)	27.88-1.69 (1.75-1.69)	27.34-2.40 (2.49-2.40)
No. of unique reflections	7728 (1223)	35211 (5545)	12572 (1970)
Completeness (%)	99.7 (99.8)	99.6 (99.3)	99.9 (100.0)
R_{merge} (%)	21.7 (82)	11.4 (90.5)	22.7 (92.3)
Mean $I/\sigma I$	8.55 (3.53)	15.16 (2.22)	9.87 (2.54)
$CC_{1/2}$	98.6 (78.6)	99.9 (85.2)	99.5 (82.9)
Redundancy	7.3 (7.4)	13.4 (13.1)	13.0 (10.7)
Wilson B factors (Å^2)	42.84	21.54	33.11
**Refinement**			
Resolution (Å)	54.32-2.83	27.88-1.69	27.79-2.40
No. of reflections used	7728	35183	12549
R_{work} / R_{free}	22.95/25.34	18.57/20.86	19.91/25.07
No. atoms			
Protein	2362	2385	2375
Ligand/ion	35	35	35
Water	40	288	91
B-factors (Å^2)			
Protein	39.59	23.19	33.36
Ligand/ion	38.35	20.31	29.34
Water	40.35	34.94	35.06
R.m.s. deviations			
Bond lengths (Å)	0.002	0.0005	0.003
Bond angles (°)	0.47	0.83	0.56
Ramachandran plot (%)			
Favoured (%)	97.37	99.01	97.70
Allowed (%)	2.63	0.99	2.30
Outliers (%)	0	0	0

	M^{pro} (P132H) -ensitrelvir	M^{pro} (G15S) -ensitrelvir	M^{pro} (K90R) -ensitrelvir
	PDB ID: 8HOM	PDB ID: 8INX	PDB ID: 8INY
**Data Collection**			
Space group	*C*2	*C*2	*C*2
Wavelength (Å)	0.9537	0.9537	0.9537
Cell dimensions			
a, b, c (Å)	97.02, 81.493, 51.545	97.75, 83.137, 51.632	96.351, 82.076, 51.476
α, β, γ (°)	90, 114.609, 90	90, 115.139, 90	90, 114.704, 90
Resolution (Å)	29.25-1.56 (1.65-1.56)	29.68-1.66 (1.72-1.66)	29.26-1.59 (1.65-1.59)
No. of unique reflections	52134 (8318)	43904 (6954)	49187 (7810)
Completeness (%)	99.5 (98.6)	99.5 (98.7)	99.5 (98.7)
R_{merge} (%)	4.2 (61.7)	4.5 (95.0)	4.5 (58.1)
Mean $I/\sigma I$	21.68 (2.83)	19.48 (1.52)	20.00 (2.71)
$CC_{1/2}$	100 (92.8)	100.0 (89.7)	99.9 (94.3)
Redundancy	6.9 (6.7)	6.9 (6.6)	6.9 (6.6)
Wilson B factors (Å^2)	23.85	30.50	26.52
**Refinement**			
Resolution (Å)	29.25-1.56	29.68-1.66	29.30-1.59
No. of reflections used	52108	43863	48782
R_{work} / R_{free}	17.13/19.62	19.78/22.20	19.24/21.39
No. atoms			
Protein	2330	2343	2352
Ligand/ion	37	37	37
Water	167	219	192
B-factors (Å^2)			
Protein	33.64	38.72	35.64
Ligand/ion	27.19	34.98	29.70
Water	40.17	45.30	42.77
R.m.s. deviations			
Bond lengths (Å)	0.005	0.006	0.005
Bond angles (°)	0.88	0.91	0.90
Ramachandran plot (%)			
Favoured (%)	97.66	98.33	98.66
Allowed (%)	2.34	1.67	1.34
Outliers (%)	0	0	0

[a] Values in parentheses are for the highest-resolution shell.

[b] $R_{merge} = \sum_h \sum_i | I_{ih} - \langle I_h \rangle | / \sum_h \sum_i \langle Ih \rangle$, where $\langle I_h \rangle$ is the mean intensity of the observations of I_{ih} of reflection h.

[c] $CC_{1/2}$: percentage of correlation between intensities from random half-datasets.

[d] $R_{work} = \sum_h | F_o - F_c | / \sum_h F_o$, where F_o and F_c are the observed and calculated structure factor amplitudes of reflection h. R_{free} is mathematically equivalent to R_{work} but was measured over 5% of the data.

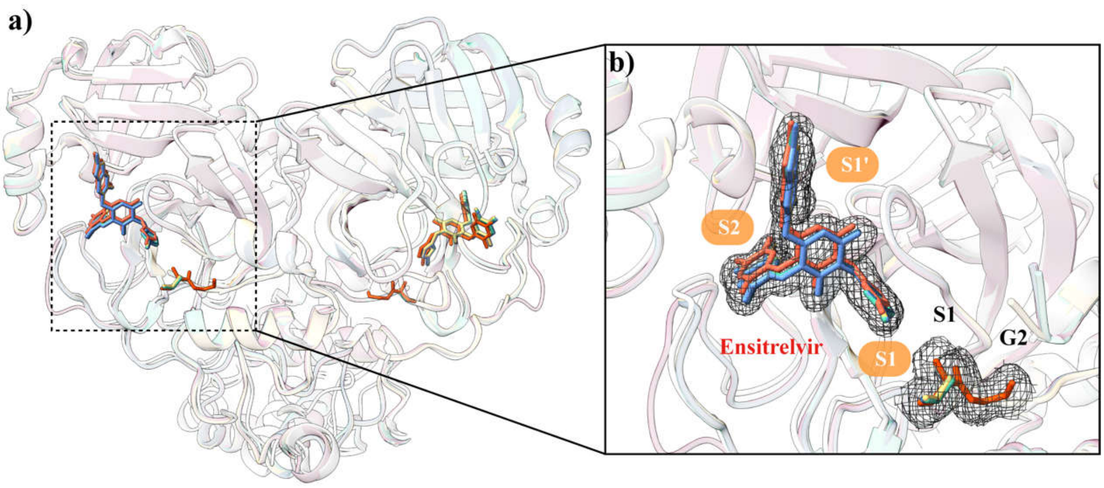

Fig. S1. Superimposed structures of WT M^{pro} and variants complexed with ensitrelvir in the dimer. **a)** WT, G15S, K90R, and P13H are respectively depicted in cartoon models, highlighted in silvery, orange red, green, and blue. The cartoon model is added with a transparency of 90, dashed boxes indicate key residues within the substrate pocket. Ensitrelvir, See1, and Gly2 are indicated in stick representation. **b)** Local zoomed-in view of the substrate pocket. The 2*Fo-Fc* density map is shown as a black grid contour at 1.0σ.

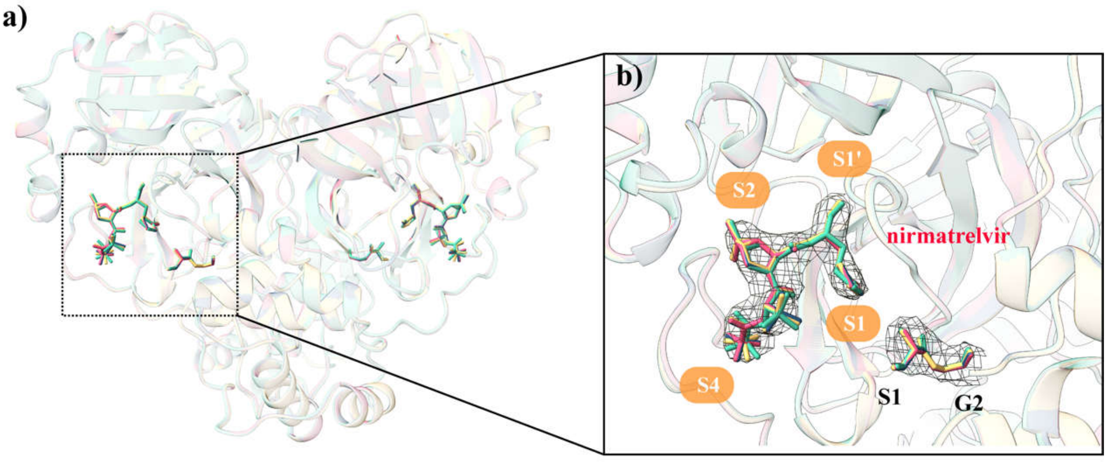

Fig. S2. Superimposed structures of WT M^{pro} and variants complexed with ensitrelvir in the dimer. **a) WT,** G15S, K90R, and P13H are respectively depicted in cartoon models, highlighted in silvery, pink, green, and yellow. The cartoon model is shown with a transparency of 90, dashed boxes indicate key residues within the substrate binding pocket. Nirmatrelvir, serine, and glycine are indicated in stick representation.

b) Local zoomed-in view of the substrate pocket. The 2*Fo*-*Fc* desntiy map is shown as a black grid contour at 1.0σ.

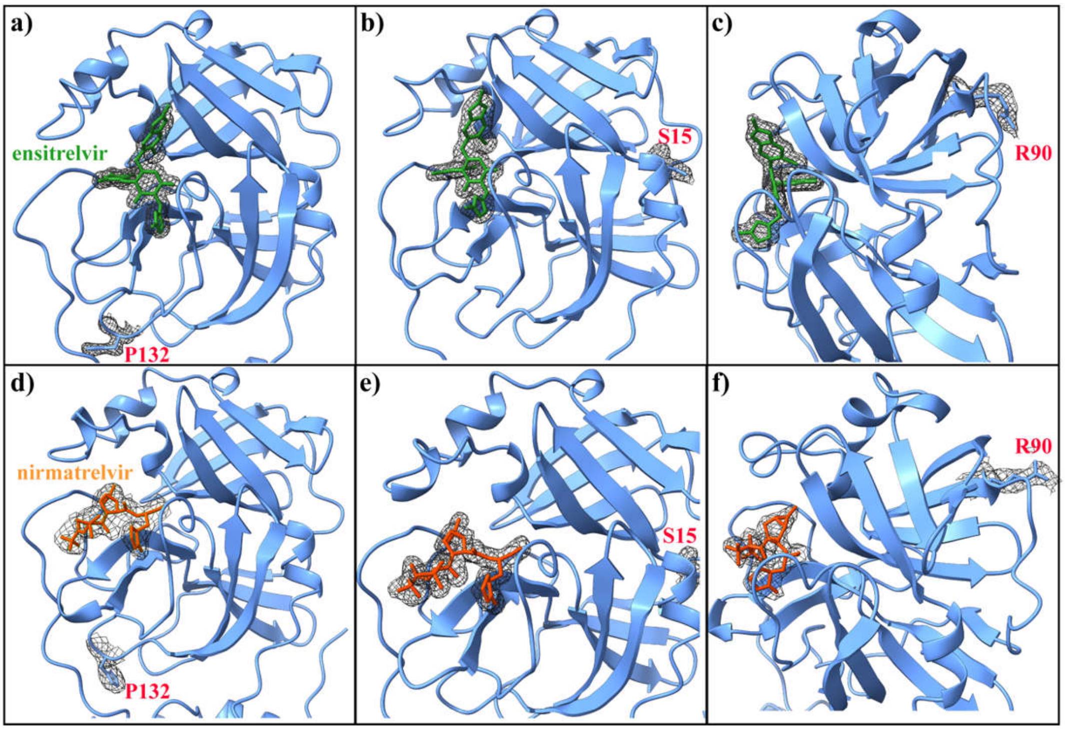

Fig. S3. 2*Fo*-*Fc* density map of M^{pro}-compound complex structures. M^{pro} mutants are shown as blue ribbon diagrams, mutated residues are shown in stick representation. Nirmatrelvir is shown as orange sticks and ensitrelvir is shown in green. The map contoured at 1.0σ is shown as black grid.

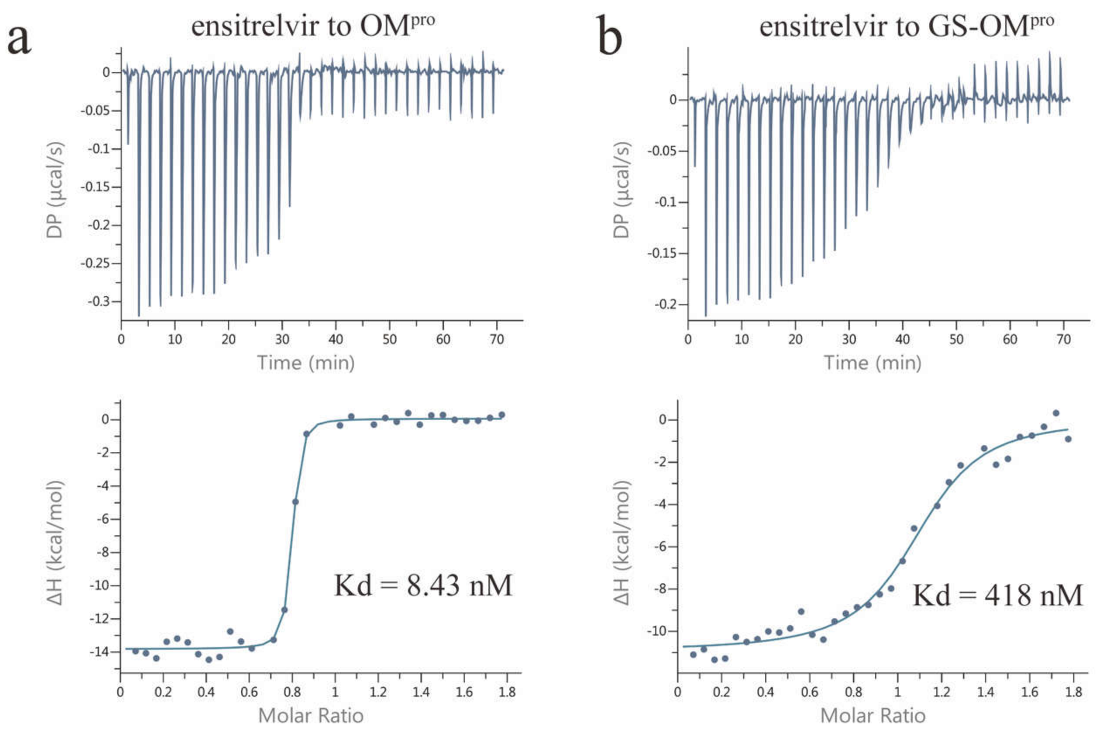

Fig. S4. Affinity testing of ensitrelvir with M^{pro} and GS-M^{pro}. ITC titrations into ensitrelvir of a) M^{pro} (Kd = 8.43 nM) and b) GS-M^{pro} (Kd= 418 nM). The upper panel shows raw heat signals, while the bottom panel shows the integrated heat and fit using a one-site binding model.
