# Supplementary material for: Molecular mechanism of ensitrelvir inhibiting SARS-CoV-2 main protease and its variants
Source: Commun Biol. 2023 Jul 5;6:694. doi: 10.1038/s42003-023-05071-y (PMC10322880; doi:10.1038/s42003-023-05071-y)
Supplement: Supplementary file 2 — Description of Additional Supplementary Files [file 42003_2023_5071_MOESM2_ESM.pdf]

Description of Additional Supplementary Files

File name: Supplementary Data 1

Description: The source data behind Figures 1a, 2f, and 3a,c in the paper.
